# Automatic Aortic Valve Cusps Segmentation from CT Images Based on the Cascading Multiple Deep Neural Networks

**DOI:** 10.3390/jimaging8010011

**Published:** 2022-01-14

**Authors:** Gakuto Aoyama, Longfei Zhao, Shun Zhao, Xiao Xue, Yunxin Zhong, Haruo Yamauchi, Hiroyuki Tsukihara, Eriko Maeda, Kenji Ino, Naoki Tomii, Shu Takagi, Ichiro Sakuma, Minoru Ono, Takuya Sakaguchi

**Affiliations:** 1 Research and Development Center, Canon Medical Systems Corporation, 1385 Shimoishigami, Otawara 324-8550, Japan; takuya2.sakaguchi@medical.canon; 2 Research and Development Center, Canon Medical Systems (CHINA) CO., LTD., Chao Yang District, Beijing 100015, China; longfei1.zhao@cn.medical.canon (L.Z.); shun.zhao@cn.medical.canon (S.Z.); xiao1.xue@cn.medical.canon (X.X.); yunxin1.zhong@cn.medical.canon (Y.Z.); 3 The University of Tokyo Hospital, 7-3-1 Hongo, Bunkyo-ku, Tokyo 113-8655, Japan; hyamauchi-tky@umin.ac.jp (H.Y.); tsukihara-circ@umin.ac.jp (H.T.); emaeda-tky@umin.ac.jp (E.M.); ino-rac@h.u-tokyo.ac.jp (K.I.); ono-tho@h.u-tokyo.ac.jp (M.O.); 4 School of Engineering, The University of Tokyo, 7-3-1 Hongo, Bunkyo-ku, Tokyo 113-8654, Japan; naoki_tomii@bmpe.t.u-tokyo.ac.jp (N.T.); takagi@mech.t.u-tokyo.ac.jp (S.T.); sakuma@bmpe.t.u-tokyo.ac.jp (I.S.)

**Keywords:** segmentation, seep learning, computed tomography, aortic valve

## Abstract

Accurate morphological information on aortic valve cusps is critical in treatment planning. Image segmentation is necessary to acquire this information, but manual segmentation is tedious and time consuming. In this paper, we propose a fully automatic aortic valve cusps segmentation method from CT images by combining two deep neural networks, spatial configuration-Net for detecting anatomical landmarks and U-Net for segmentation of aortic valve components. A total of 258 CT volumes of end systolic and end diastolic phases, which include cases with and without severe calcifications, were collected and manually annotated for each aortic valve component. The collected CT volumes were split 6:2:2 for the training, validation and test steps, and our method was evaluated by five-fold cross validation. The segmentation was successful for all CT volumes with 69.26 s as mean processing time. For the segmentation results of the aortic root, the right-coronary cusp, the left-coronary cusp and the non-coronary cusp, mean Dice Coefficient were 0.95, 0.70, 0.69, and 0.67, respectively. There were strong correlations between measurement values automatically calculated based on the annotations and those based on the segmentation results. The results suggest that our method can be used to automatically obtain measurement values for aortic valve morphology.

## 1. Introduction

Heart valve disease is one of the most common causes of heart failure, which affects approximately 13.2% of people over the age of 75 [1], and the number of patients with valvular disease is increasing due to the global population aging. More than 250,000 heart valve operations are performed each year in the world [2]. In order to make an operative plan containing procedure and device selections, it is important to obtain accurate information about the valve morphology. In particular, the acquisition of valve morphology based on CT images, which has high spatial resolution and provides information on the relationship with surrounding structures, is one of the most important clinical workflows in current clinical situations for treatment planning in aortic valve disease [3]. However, acquiring information on valve morphology from CT images is a difficult and time consuming task for physicians and radiological technologists.

There are several products available to support the acquisition of valve morphology, however most require multiple manual operations by the user, resulting in increased work for the user and variability among users. In order to eliminate these manual operations, it is necessary to have a technique to appropriately segment the target anatomical structures from medical images. Many methods have been reported for segmenting anatomical structures from medical images [4,5,6,7,8,9]. In addition, there have been many reports on segmentation methods using machine learning [10,11]. However, since the aortic valve is a very thin and fluttering structure, it is expected that simply applying segmentation methods proposed for other structures to the aortic valve cusps will not provide accurate segmentation. There are previous studies that have proposed segmentation algorithms for aortic valves, however most do not perform segmentation for valve cusps [12,13,14]. Pouch et al. proposed an algorithm for valve cusps segmentation from 3D transesophageal echocardiographic images, however this algorithm is a semi-automatic method that requires user input [15]. Fan et al. proposed a deep learning-based algorithm to segment each cusp from CT images, although it required input images accurately cropped around the valve. In addition, the accuracy verification for cases with severe calcification is insufficient [16]. Pak et al. proposed a deep learning-based automatic segmentation method for aortic valve cusps from CT images [17]. They transform the original CT image into a sub-region containing the aortic valve using Spatial Transformer Network (STN) before segmentation processing for computational efficiency. However, the transformed sub-region may sometimes omit parts of the aortic root and affect the performance. In addition, the impact of their automatic segmentation on measurements that are important for surgical planning, have not been verified.

The contributions of this work are summarized as follows.

1. To the best of our knowledge, this is the second study to build a fully automatic method for segmenting aortic valve cusps. Our method constructs a computational flow of cascaded multiple networks for landmark detection and segmentation without transforming the original images. This is expected to improve performance as the input size for the networks is reduced step by step and sub-regions are more accurately located. Each network is based on methods presented in previous studies [18,19], however the method of combining them is reported for the first time.
2. We proposed an evaluation method for the impact of segmentation results on measurement values for each measurement item, and evaluated our proposed segmentation method using this evaluation method. Many papers on segmentation methods focus on segmentation accuracy and do not investigate the impact on measurements. To the best of our knowledge, this paper is the first work to investigate the impact of aortic valve cusps segmentation results on various measurements.

This paper is organized into five sections. In Section 2, which is the Materials and Methods section, Section 2.1 describes the datasets we used in detail. Section 2.2 explains our proposed segmentation method in detail, and Section 2.3 describes the evaluation method for the proposed segmentation method. Evaluation results are presented in Section 3, showing the segmentation accuracy of the proposed method and its impact on each measurement item. In Section 4, we discuss the evaluation results. Section 5 presents a conclusion for our work.

## 2. Materials and Methods

### 2.1. Data and Annotation

ECG gated cardiac 3D- or 4D- CT imaging data from 138 patients were retrospectively collected at multiple clinical institution with the approval of the institutional review boards. These patients presented with various cardiac diseases including stenotic aortic valve, regurgitant aortic valve and other heart diseases with normal aortic valve, however not containing bicuspid valves. The CT images were acquired with contrast using Aquilion ONE (Canon Medical Systems Corporation, Otawara, Tochigi, Japan) or SOMATOM Forc (SIEMENS, Munich, Germany). The ECG gated cardiac CT sequences include 1–20 volumes per cardiac cycle, where each volume contains 172–667 slices with 512 × 512 pixels. The in-slice resolution is isotropic with 0.311–0.625 mm and the slice thickness is 0.5 to 0.75 mm.

The eight ground-truth landmarks and the four ground-truth labels (GT) were annotated. The eight ground-truth landmarks are 3D coordinates of the three Nadir points, which are defined as points on the cusp that are on the most left ventricular outflow tract side for each of the right-, left- and non-coronary cusps (RCC, LCC and NCC), the three commissure points and the two coronary ostium points, which are defined as the proximal edge of the ostium, for the left and right coronary ostium. The four ground-truth labels are a set of pixels corresponding to each region of the aortic root and each cusp.

The annotation process was performed manually or semi-automatically according to the following four steps by physicians, radiological technologists or general technologists under the instruction of an expert physician, and all annotations were checked and approved by the expert physician using in-house software.

1. Volume data in end systolic (ES) and/or end diastolic (ED) phases suitable for acquiring morphological information of the aortic valve were selected for each CT imaging data based on the information available from CT images, which included ECG gating timing, LV volume size, aortic valve motion and blurring. A total of 258 volume data were selected in this step, and the following annotation work was performed only on these volume data.
2. The Vitrea workstation (Canon Medical Informatics, inc., Otawara, Tochigi, Japan) was used to semi-automatically generate an initial label of the aortic root by providing seed points within the aortic root region. The initial label was manually modified as needed.
3. Each of the eight landmarks were manually annotated one point on CT image.
4. The labels for each cusp were separately manually annotated on the internal regions of the aortic root. Each cusp did not have an overlapping area.

### 2.2. Aortic Valve Cusps Segmentation Method

**Figure 1:**
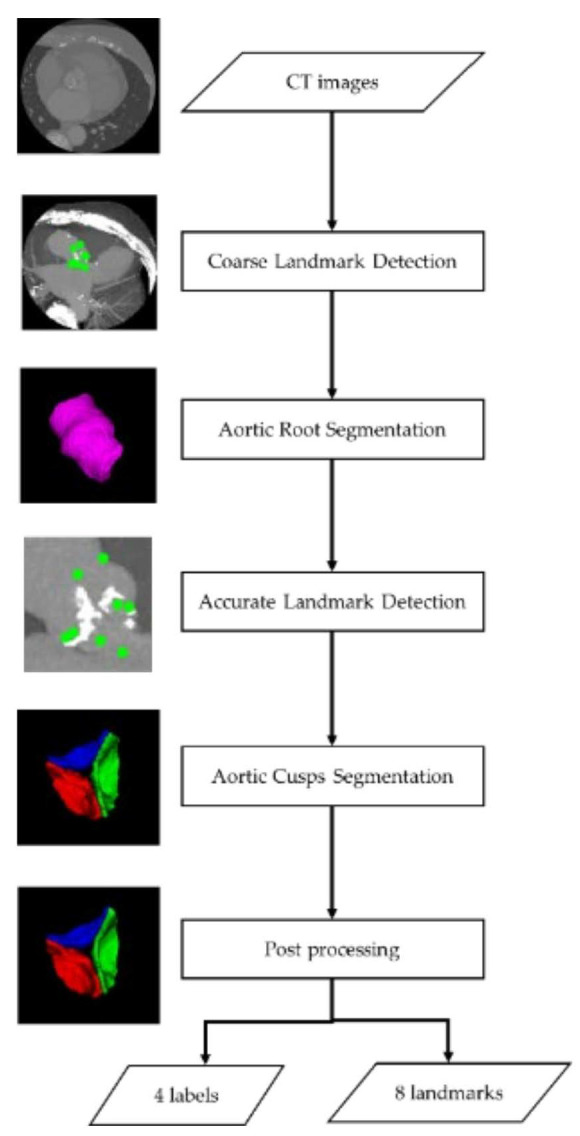
Flow chart of segmentation method.

Our segmentation method contains five primary steps (Figure 1): (1) detect eight landmarks coarsely; (2) segment the aortic root within the cropped regions based on coarse landmarks; (3) detect eight landmarks accurately within the cropped region based on the aortic root region; (4) segment aortic cusps within the cropped region based on the aortic root region; and (5) post-process for aortic cusps labels.

#### 2.2.1. Landmark Detection Processing

Resampling for original CT images was performed as preprocessing for input images of landmark detection processing by using SimpleITK (version 1.2.4). In the resampling, original CT images were cropped with fixed pixel spacing and matrix size. If the cropped region included the outer region of original CT images, the pixel values of the outer region were padded with −3024. For the coarse landmark detection, original CT images were resampled into isotropy-based images with pixel spacing of 1.5 mm and matrix and size 128 × 128 × 128 around the center of the original CT images. For the accurate landmark detection, original CT images were resampled into isotropy-based images with pixel spacing 0.5 mm and matrix size 128 × 128 × 128 around the center of the bounding rectangular region of the segmented aortic root region.

**Figure 2:**
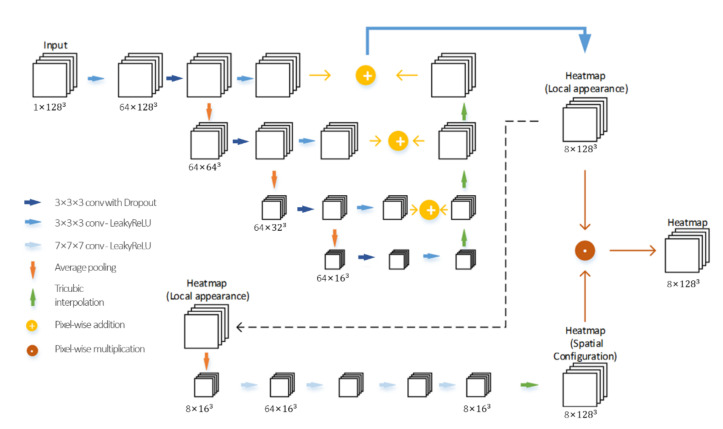
SCN architecture for landmark detection.

3D Spatial Configuration-Net (SCN) [18] with updated parameters was used as our network architecture for both coarse and accurate landmark detection (Figure 2). The loss function (1) proposed by Payer et al. [18] was used with parameter α = 50 and λ = 0.0005,

(1) $$\underset{w,b,\sigma }{\min}\overset{N}{\underset{i=1}{\sum }}\underset{x}{\sum }||h_{i}\left(x;w,b\right)-g_{i}\left(x;\sigma_{i}\right){||}_{2}{}^{2}+\alpha ||\sigma {||}_{2}{}^{2}+\lambda ||w{||}_{2}{}^{2}$$

minimized using the Adam optimizer with a learning rate 5 × 10^−5^, β_1_ = 0.9 and β_2_ = 0.999 empirically. We used the mini-batch size of 1. The number of training iterations was 200,000. Tensorflow (version 1.13.1) was used for training and testing of the network.

We introduced on-the-fly data augmentation using SimpleITK (version 1.2.4). Intensity values are randomly multiplied by [0.75, 1.25] and shifted by [−0.25, 0.25]. The image is randomly translated, rotated, and scaled. In addition, elastic deformation is employed by randomly shifting points on a regular grid of 15 × 15 pixels by 5 pixels and interpolating with a cubic B-spline. All scaling operations are randomly sampled from a uniform distribution within a specified interval.

#### 2.2.2. Segmentation Processing

The aortic root segmentation classifies voxels into two classes: background and aortic root. The aortic cusps segmentation classifies voxels into four classes: background, LCC, RCC, and NCC. Pytorch (version 1.6.0) was used for training and testing of the network.

Resampling and normalizing for the original CT images were performed as preprocessing for input images of segmentation processing by using interpolation method from Pytorch (version 1.6.0). For the aortic root segmentation, the original CT images were cropped with the rescaled region 1.5 times larger than the bounding rectangular region of the detected coarse landmarks, resampled into isotropy-based images with the pixel spacing 0.4 mm and same physical matrix size as the cropped CT images, and normalized by z-score based on the mean and standard deviation of all intensity values collected from the training datasets. For the aortic cusps segmentation, original CT images were cropped with the bounding rectangular region of the segmented aortic root region, resampled into isotropy-based images with the pixel spacing 0.4 mm and same physical matrix size, and normalized as in the aortic root segmentation.

**Figure 3:**
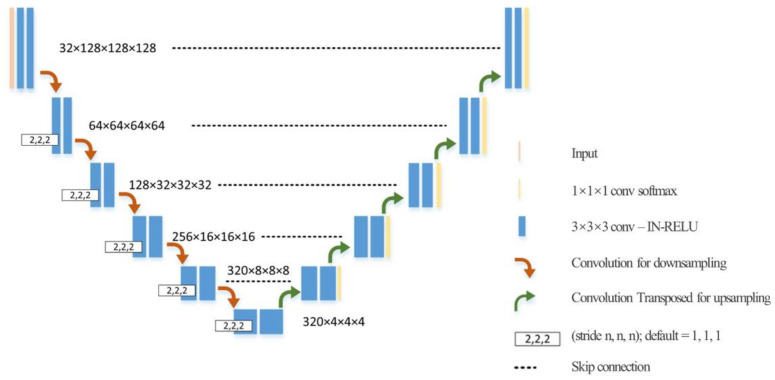
U-Net architecture for segmentation.

3D full resolution U-Net [19] containing five sampling layers and five up sampling layers were used as the network architecture for both aortic root and aortic cusps segmentation processing (Figure 3). Patch size was 128 × 128 × 128. To handle varying volume sizes from preprocessed images, a sliding window algorithm was used. The full volume was split into blocks based on the patch size, and the neural network predicted on each block. Default loss function, which combines cross entropy loss and soft dice loss, were used for training U-Net. The training epoch was 1000, and batch size was 2. Stochastic gradient descent with nesterov momentum (μ = 0.99) and an initial learning rate of 10^−2^ was used for earning network weights. Other parameters of 3D full resolution U-Net were the same as in Isensee et al. [19].

We use the default method to perform on-the-fly data augmentation. The images are randomly rotated by (−30°, 30°). The intensity values are randomly multiplied with (0.7, 1.4). Other data augmentation is same as in [19].

#### 2.2.3. Post-Processing

All regions except for the region with largest volume were removed if the segmentation process resulted in multiple independent regions for a single component. If there were any holes in the segmentation results, the holes were filled based on the method of Aktouf et al. [20]. The insertion line of the cusp was extended to always connect with the vessel wall inside the Sinus of Valsalva if it was not connected to the vessel wall.

### 2.3. Evaluation

#### 2.3.1. Evaluation Setup

The 258 CT volume data were split approximately 6:2:2 for the training, validation and test steps, respectively, taking into account data collection site, cardiac phase (ED or ES) and calcium condition (with severe calcification or without severe calcification). Stratified five-fold cross validation was performed to evaluate the accuracy of the landmark detection and segmentation processes.

#### 2.3.2. Automation and Processing Time

The segmentation results of the test steps for all cases were visually checked by both Multiplanar Reconstruction (MPR) images overlaid with the segmentation results and the Volume Rendering (VR) images created based on the segmentation results. VR images were overlaid with regions of CT values above the user selected threshold (500–1000 HU) within the aortic root as the calcium region. In addition, the total-processing times on a PC with an Intel Xeon Sliver4214 processor and an NVIDIA GeForce RTX 2080 SUPER were also recorded.

#### 2.3.3. Landmark Evaluation

The accuracy of the landmark detection processing was evaluated by the Euclidean distance between the ground-truth landmarks and the landmark detection results for each of the eight landmarks. For each coarse and accurate landmark detection processing, this evaluation was performed on each of the CT volumes with four different valve conditions including ES phase with severe calcification, ED phase with severe calcification, ES phase without severe calcification and ED phase without severe calcification.

#### 2.3.4. Segmentation Evaluation

The accuracy of the segmentation processing was evaluated by comparing the ground-truth label (*G*) with the segmentation result (*S*) using the following three evaluation measures for each of the four valve components (aortic root and each cusp). These evaluations also were performed on each of four different CT volumes. The aorta root segmentation was evaluated only for the area between the Nadir plane, which is defined as the plane passing through the three Nadir points, and the sinotublar junction plane defined by the expert, in order to make an accurate assessment for the Sinus of Valsalva region.

1. Volumetric overlap: Dice Coefficient (DC) [21] was calculated as volumetric overlap between two sets of voxels G and S, which was defined as follows:

(2) $$\mathrm{DC}\left(G,\;S\right)=2\left|G\cap S\right|/\left(\left|G\right|+\left|S\right|\right)$$

2. Surface Distance: The Mean symmetric Surface Distance (MSD) is defined as follows:

(3) $$\mathrm{MSD}\left(G,\;S\right)=\frac{1}{\left|S\left(G\right)\right|+\left|S\left(S\right)\right|}\left(\underset{s_{G}\in S\left(G\right)}{\sum }d_{m}\left(s_{G},\;S\left(S\right)\right)+\underset{s_{S}\in S\left(S\right)}{\sum }d_{m}\left(s_{S},\;S\left(G\right)\right)\right)$$

where *d_m_*(,) denotes the minimum Euclidean distance between an arbitrary voxel to an arbitrary set of surface voxels, *S*(*G*) denotes the set of surface voxels of *G*, *s_G_* denotes each voxel of *S*(*G*), *S*(*S*) denotes the set of surface voxels of *S*, *s_S_* denotes each voxel of *S*(*S*).

In addition, Hausdorff Distance (HD) was also calculated as follows:

(4) $$HD\left(G\;,\;S\right)=\underset{s_{S}\in S\left(S\right)}{\max}\left\{d_{m}\left(s_{S},\;S\left(G\right)\right)\right\}$$

**Figure 4:**
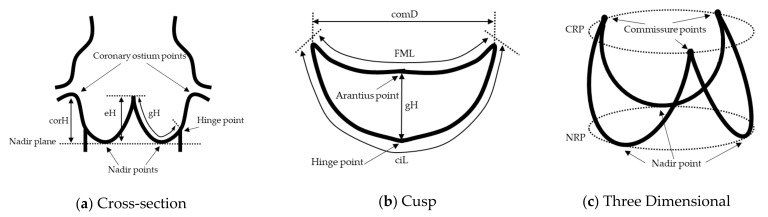
Schematic diagram illustrating the measurement position. (**a**) Cross-section, (**b**) Cusp, (**c**) Three Dimensional.

3. Impact on measurements: For the eight measurement items, the differences between the values measured based on *G* and measured based on *S* were calculated using the same measurement algorithm, as the impacts for each measurement item (Figure 4). Each measurement item was defined in this paper as follows:

- effective Height (eH): Straight line distance from Arantius body to Nadir plane;
- geometric Height (gH): Shortest curved line distance along the surface of the cusp, from Arantius body to Hinge point;
- cusp insertion Length (ciL): Curved line distance between commissure points on the contour of the cusp in contact with the Sinus of Valsalva;
- Free Margin Length (FML): Curved line distance between commissure points on the contour of the cusp not in contact with the Sinus of Valsalva;
- commissural Distance (comD): Straight line distance between commissure points;
- Nadir Ring Perimeter (NRP): Perimeter of the contour of the Aortic root region on the Nadir plane;
- Commissure Ring Perimeter (CRP): Perimeter of the contour of the Aortic root region on the Commissure plane;
- coronary Height (corH): Straight line distance from coronary ostium point to Nadir plane.

Arantius body is defined as the middle point on FML. Hinge point is defined as points on ciL that is most on the left ventricular outflow tract side for each cusp. In many cases, Hinge point is the same position as Nadir point.

#### 2.3.5. Statistical Analysis

Continuous data was given as mean and standard deviation (± SD) for cases. The relation between measurement vales based on *G* and *S* was analyzed by Pearson’s product–moment correlation coefficient (*r*), scatterplot and the Bland–Altman difference plot with 1.96 SD intervals. For each measurement value error, the absolute error and error rate were also presented as mean and standard error (SE). The fixed errors between the measurement values based on *G* and *S* were analyzed by the paired-*t* test. The proportional errors were analyzed by Pearson’s product–moment correlation coefficient (*r_p_*) between the difference and the mean between measurement values based on *G* and *S.* IBM SPSS Statistics (version 26.0, IBM, New York, NY, USA) was used for all statistical analysis. In this study, Bonferroni correction was used to adjust for multiple validation endpoint in the 19 measurement items, and significant differences were considered if *p* < 0.0026.

## 3. Results

**Figure 5:**
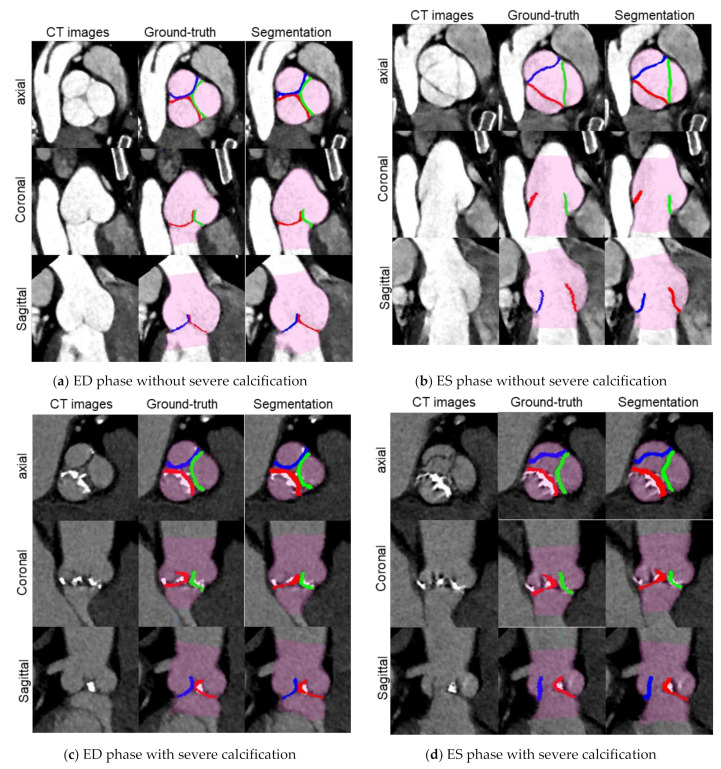
Visualization of CT images (WW/WL: 650/300) only, overlaid with each ground-truth label and overlaid with each segmentation result in the test step for 2 cases: (**a**,**b**) a case without calcification; (**c**,**d**) a case with severe calcification. Translucent purple: aortic root labels, Green: RCC labels, Blue: LCC labels and Red: NCC labels.

**Figure 6:**
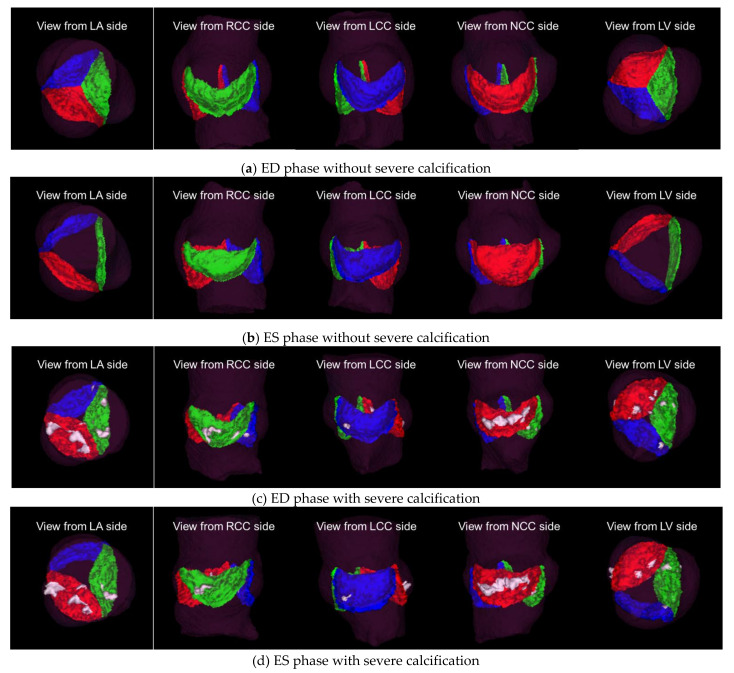
Visualization from five directions of VR images of segmentation results for two of the same cases as Figure 4: (**a**,**b**) a case without calcification; (**c**,**d**) a case with severe calcification. Translucent purple: aortic root labels, Green: RCC labels, Blue: LCC labels, Red: NCC labels and White: Calcium.

A total of 258 volume data were selected from 138 CT imaging data, containing 87 ED without severe calcification data, 42 ED with severe calcification data, 87 ES without severe calcification data and 42 ES with severe calcification data. For all volume data, the ground truth data was created, and our proposed aortic valve cusps segmentation was performed. The segmentation was successful for all volume data with 69.26 ± 7.42 s as mean total-processing time. The morphology conditions of the cusps are different depending on the cardiac phase and amount and distribution of calcium, however our algorithm was able to generally segment all cusps in each condition (Figure 5 and Figure 6). These MPR and VR images provide visual information about the aortic valve morphology and the distribution of calcification on each cusp.

Table 1 shows the results of five-fold cross validation for each coarse and accurate landmark detection for each of the valve conditions. Coarse and accurate landmark detection showed an error of around 1.0 to 2.0 mm for each landmark in each image condition. Accuracies were improved overall with accurate landmark detection over coarse landmark detection. The accuracy tended to be higher in cases with severe calcifications than in cases without severe calcifications, except for Nadir in LCC in the ED phase and Nadir in NCC in the ES phase. There was no overall trend in the detection accuracy due to the difference in phase.

Table 2 shows the results of five-fold cross validation for Dice Coefficient (DC), Mean symmetric Surface Distance (MSD) and Hausdorff Distance (HD) for each valve conditions. In the accuracy of each cusps, DC was around 0.7, MSD was 0.34 to 0.45 mm, and HD was around 1 mm. DC tended to be lower in NCC in cases with severe calcifications. The MSD and HD tended to be smaller overall in ED phase than in ES phase. There was no overall trend in the segmentation accuracy with or without severe calcifications.

**Figure A1:**
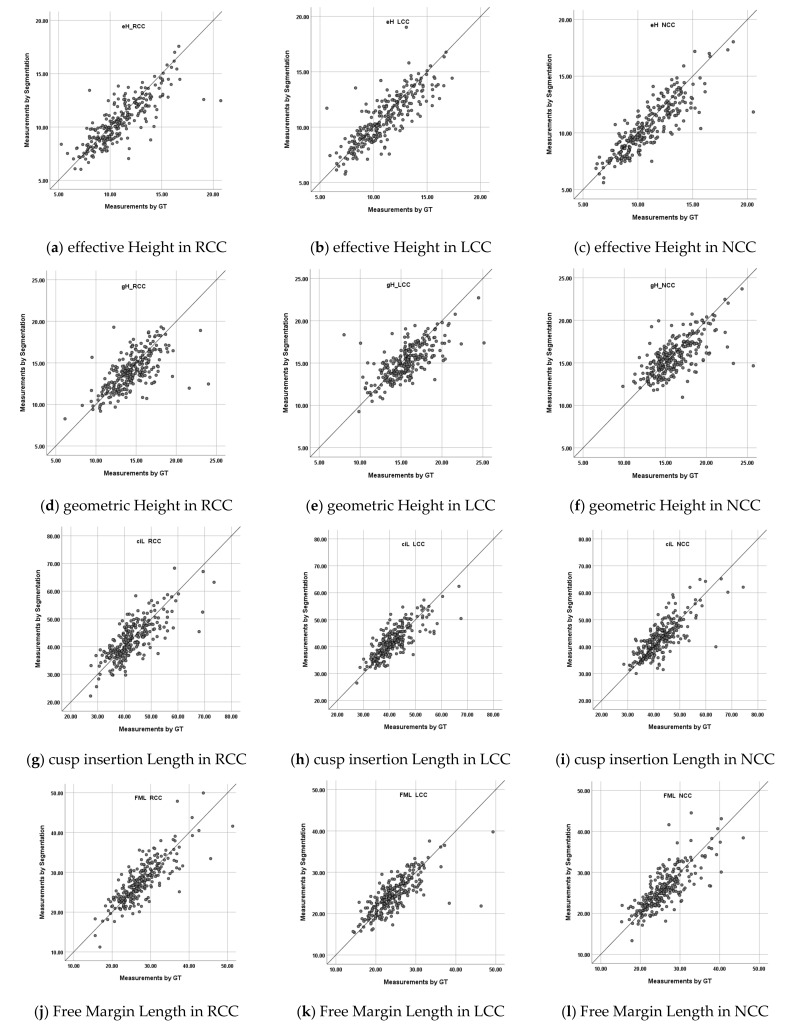

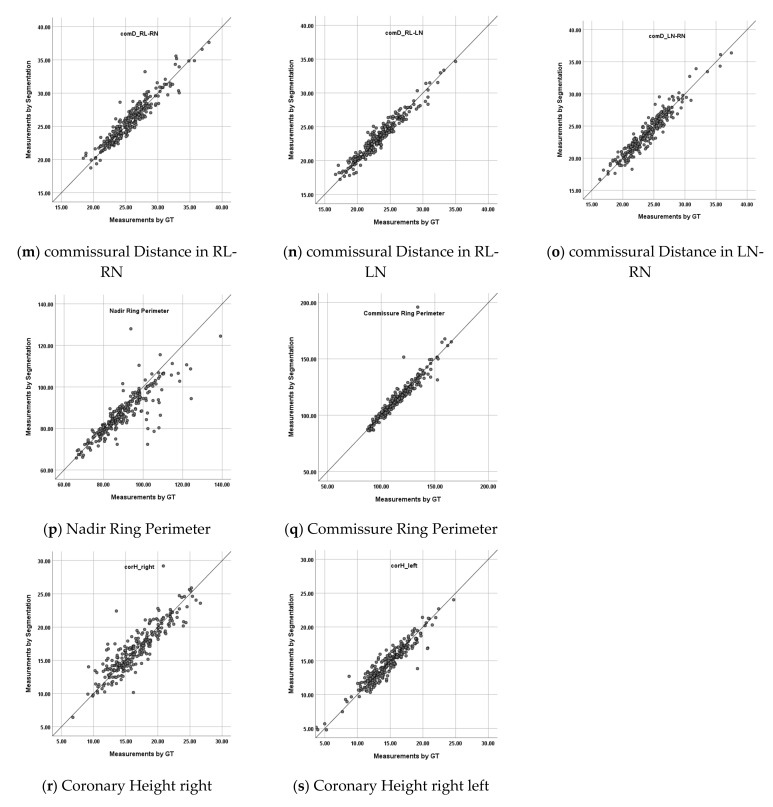
Scatterplots for correlation for each measurement item: (**a**) effective Height in RCC, (**b**) effective Height in LCC, (c) effective Height in NCC, (**d**) geometric Height in RCC, (**e**) geometric Height in LCC, (**f**) geometric Height in NCC, (**g**) cusp insertion Length in RCC, (**h**) cusp insertion Length in LCC, (**i**) cusp insertion Length in NCC, (**j**) Free Margin Length in RCC, (**k**) Free Margin Length in LCC, (**l**) Free Margin Length in NCC, (**m**) commissural Distance in RL-RN, (**n**) commissural Distance in RL-LN, (**o**) commissural Distance in LN-RN, (**p**) Nadir Ring Perimeter, (**q**) Commissure Ring Perimeter, (**r**) Coronary Height right, (**s**) Coronary Height right left.

**Figure A2:**
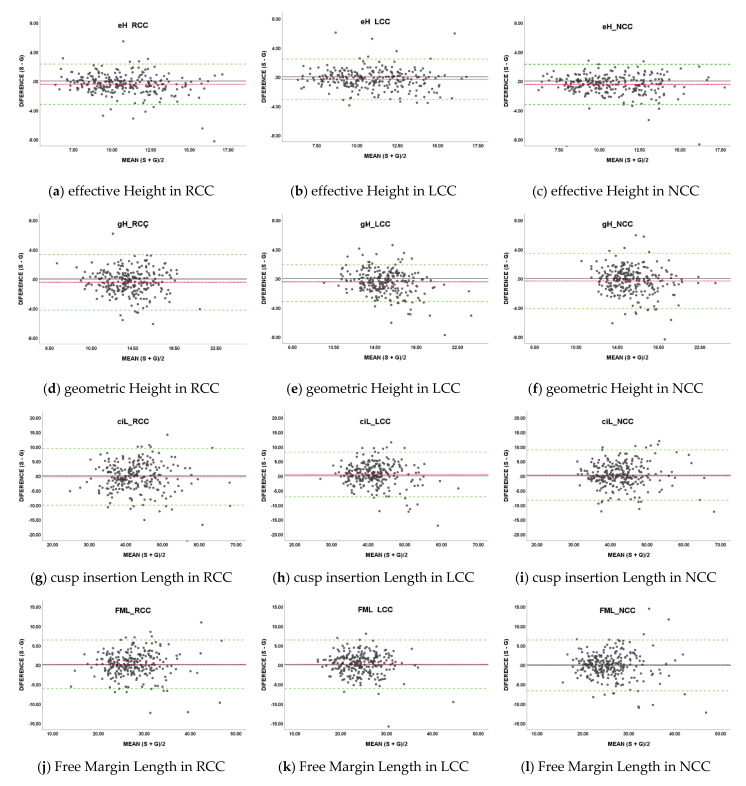

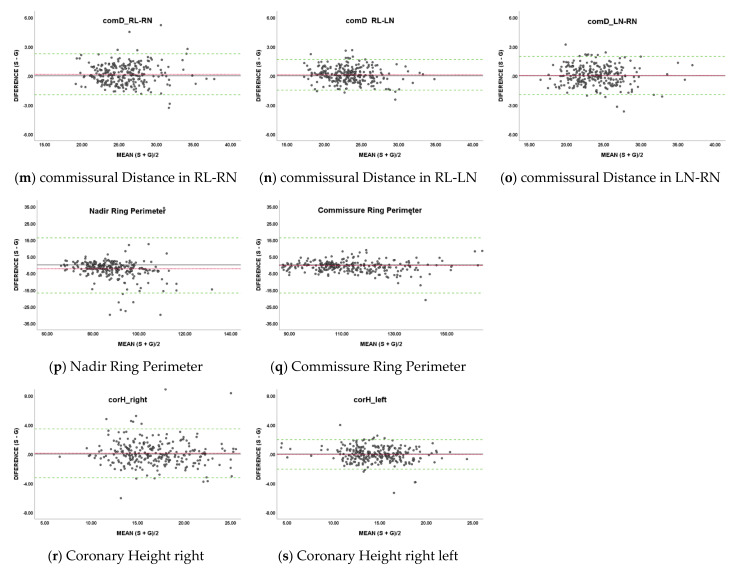
Bland–Altman plots for each measurement item: (**a**) effective Height in RCC, (**b**) effective Height in LCC, (**c**) effective Height in NCC, (**d**) geometric Height in RCC, (**e**) geometric Height in LCC, (**f**) geometric Height in NCC, (**g**) cusp insertion Length in RCC, (**h**) cusp insertion Length in LCC, (**i**) cusp insertion Length in NCC, (**j**) Free Margin Length in RCC, (**k**) Free Margin Length in LCC, (**l**) Free Margin Length in NCC, (**m**) commissural Distance in RL-RN, (**n**) commissural Distance in RL-LN, (**o**) commissural Distance in LN-RN, (**p**) Nadir Ring Perimeter, (**q**) Commissure Ring Perimeter, (**r**) Coronary Height right, (**s**) Coronary Height right left. Red dashed lines indicate the mean of the difference, the Green dashed lines indicate the 1.96 SD intervals as the limits of agreement.

Table 3 shows the results of five-fold cross validation for the difference of measurement values, and Figure A1 shows scatterplots for correlation and Figure A2 shows Bland–Altman plots. Significant correlations were shown between the ground truth based-measurement values and the segmentation result based-measurement values for all measured items. The correlation coefficients were above 0.7 except for geometric Heights, indicating a strong correlation. The absolute errors were less than 5 mm, and the error rates were less than 10% for all the measured value items. Significant fixed errors were shown only for effective Heights, geometric Heights and the Nadir Ring Perimeter. Proportional errors were none or only weak relationships were observed.

## 4. Discussion

We proposed an automatic segmentation method for aortic valve cusps that cascades networks for landmark detection and networks for segmentation. Our proposed method was able to fully automate segmentation of the aortic valve cusps for ES and ED phases with or without severe calcifications. Segmentations can provide more visual information about the morphology of the aortic valve and the distribution of calcification. In Figure 5 and Figure 6, we can visually assess that the cusps without severe calcification (RCC, LCC and NCC in the case without severe calcification, and NCC in the case with severe calcification) are open in ES phase and closed in ED phase, while the cusps with severe calcification (RCC and LCC in the case with severe calcification) demonstrate minimal change in their position between ES and ED phases. Such visual information would be useful for treatment planning for surgical and interventional procedures.

Our method was able to detect eight landmarks with an error of about 1 to 2 mm. Considering that the absolute error of each measurement item is around 0.5 to 4.5 mm as shown in Table 3, the accuracy of landmark detection has a significant impact on the measurement accuracy, and therefore it is assumed that landmark detections are important steps in this method. The fact that the accuracy of landmark detection tended to be higher in cases with severe calcification than in cases without severe calcification may be due to the distribution of pixels with high HU values in cases with severe calcification showing the morphological characteristics of the aortic valve.

Although the segmentation accuracy cannot be directly compared with previous studies due to the different validation cases, the accuracy of our method was comparable or higher than previous studies in Dice coefficient [16,17]. We did not find a clear reason why the NCC in cases with severe calcifications showed low DC. This may perhaps be due to the fact that the image data sets used in this study included many cases with severe calcification in the NCC. The relationship between the amount and distribution of calcification and segmentation accuracy needs to be further investigated. For MSD and HD, the higher accuracy of the ED phase compared to the ES phase can be explained by the lower visualization ability for the tip of the aortic valve on CT images in the ES phase. In the ES phase, the aortic valve is in an open position and the valve tip is fluttering due to the blood flow, so it often appears as double or triple valve leaflets or blurred images on CT images. This increases the difficulty of segmentation and leads to low accuracy. As the temporal resolution of CT scanners will be improved in the future, the difference in accuracy between different phases will be insignificant.

For all measurement items, when the same measurement algorithms were used, there was a strong correlation between the measurement values calculated based on the ground truth labels and those calculated based on the segmentation results and the error rates were less than 10%. On the other hand, there were significant fixed errors for effective Heights, geometric Heights and the Nadir Ring Perimeter. Both effective Heights and geometric Heights are measurement items that depend on the location of the Arantius body, so there may be some bias in the segmentation accuracy around the Arantius body. The Nadir Ring Perimeter is a measurement item dependent on the location of nadirs and the contour of the LVOT region. There may be some bias in the segmentation accuracy of the LVOT region, as fixed errors were not found in the other measurement items dependent on the location of nadirs.

The mean absolute errors for the Nadir Ring Perimeter and Commissure Ring Perimeter were 4.39 mm and 3.31 mm, respectively. The most frequently used device in Transcatheter Aortic Valve Implantation (TAVI) currently, SAPIEN 3(Edwards Lifesciences, Irvine, CA, USA) has four different diameter sizes: 20, 23, 26, and 29 mm [22], while the Evolut^TM^ R (Medtronic plc, Minneapolis, MN, USA) also has four different sizes: 23, 26, 29 and 34 mm [23]. This means that the closest size devices differ by is 3 mm in diameter, which means a difference of about 9.42 mm in perimeter. The facts that the mean absolute errors between the ground truth based- and segmentation result based- measurement values for the Nadir Ring Perimeter and for Commissure Ring Perimeter were less than 9.42 mm may suggest that our proposed method is clinically usable.

The development of an automatic aortic valve measurement application based on our method will allow users to obtain aortic valve morphology information more quickly without inter-user variability for treatment planning. In addition, segmentation results of each valve component can also be used as input data for other techniques to assist in treatment planning, such as the creation of 3D heart valve models by a 3D printer [24,25] and simulation of valve structure and fluid flow [26].

This paper contains the following limitations.

Our method does not support cases with bicuspid valves, which occur with a frequency of about 0.8% [27], as our method always outputs three labels as cusp components. There was difficulty in collecting a sufficient number of CT images with bicuspid valves to develop the learning model that would support bicuspid valve cases. In addition, in order to support both bicuspid and tricuspid valve cases, it is necessary to develop a new algorithm that can dynamically change the number of components to be outputted depending on the target organ conditions.

Our method does not support cases with holes or cases in which the insertion line is disconnected from the vessel wall, as our post-processing modifies them. If a more accurate learning model can be developed, this problem can be solved by removing post-processing from the algorithm.

Our method is only supported for the ES and ED phases as our learning model does not learn the morphology of phases other than the ES and ED. This is the result of us not being able to create accurate GTs for these phases due to motion artifacts caused by valve movement. Segmentations for the ES and ED phases may be sufficient if only the valve morphology information is needed for treatment planning. On the other hand, it is not sufficient to obtain accurate valve movement information. If the CT scanner can be developed to take CT images without valve motion artifacts for all phases, our method will be able to support all phases by learning the valve morphology information of other phases.

The use of our method in a real clinical situation requires a manual task to identify the ES and ED phases with low valve motion artifacts. It may be possible to identify this automatically by estimating the cardiac phase using information from the DICOM header and LV volumes calculated based on previous studies [28,29], and obtaining information about valve motion artifacts using simple image processing techniques such as Hessian filter.

All GTs were approved by one physician only. There may be value in creating the GTs from multiple physicians to test whether our segmentation results fall within the range of physician variability. On the other hand, it may not be a major problem that the GT is based on only a single physician, since several studies have shown that the measurement of valve morphology on CT images before TAVI has a small interobserver variability [30,31].

The performance of our method has not been directly compared to the performance reported in previous studies using the same CT datasets. In addition, although U-Net has been used as the segmentation network in the proposed method, we have not compared its performance with other networks.

## 5. Conclusions

We developed an automatic segmentation method for aortic valve cusps from CT images using the deep learning-based algorithm that is cascaded multiple networks. Our method will provide more visual information regarding the morphology of the aortic valve, so it will support the development of a more accurate and detailed surgical plan. We also investigated the impact of segmentation results on various measurement values, which suggest that our method can be used to obtain measurement values automatically. The automatic measurement of aortic valve morphology is expected to reduce the labor required by physicians and radiological technologists for aortic valve treatment planning, and to enable more accurate and consistent treatment planning by acquiring information without user variation.

In future work, we will consider solutions to the above-mentioned limitations and conduct clinical studies, such as comparing the intraoperative direct observation and the manual measurements by physicians with the automatic measurements by our proposed method, in order to more concretely verify the clinical effectiveness and risks of the proposed method.

## Figures and Tables

**Table 1:** Landmark Detection Results.

Coarse Landmark Detection	Nadir RCC [mm]	Nadir LCC [mm]	Nadir NCC [mm]	Commissure RL [mm]	Commissure RN [mm]	Commissure LN [mm]	Coronary Ostium R [mm]	Coronary Ostium L [mm]
ES w/o sCa	2.08 ± 1.12	1.62 ± 0.93	1.68 ± 1.08	2.08 ± 1.14	2.15 ± 1.47	1.98 ± 1.23	1.61 ± 1.51	1.54 ± 1.32
ED w/o sCa	1.72 ± 0.91	1.68 ± 0.77	1.72 ± 1.11	1.84 ± 1.24	2.17 ± 1.27	1.74 ± 1.21	1.46 ± 1.41	1.37 ± 0.98
ES w/sCa	1.57 ± 0.87	1.46 ± 0.83	2.18 ± 1.43	1.34 ± 0.62	1.76 ± 1.11	1.73 ± 1.53	1.53 ± 1.16	1.31 ± 0.81
ED w/sCa	1.37 ± 0.74	2.13 ± 3.29	1.54 ± 0.99	1.34 ± 0.90	1.63 ± 1.04	1.41 ± 1.08	1.29 ± 0.84	1.33 ± 0.73
Total	1.76 ± 0.99	1.70 ± 1.54	1.75 ± 1.15	1.76 ± 1.11	2.01 ± 1.30	1.77 ± 1.26	1.49 ± 1.33	1.41 ± 1.05
**Accurate Landmark Detection**	**Nadir RCC [mm]**	**Nadir LCC [mm]**	**Nadir NCC [mm]**	**Commissure RL [mm]**	**Commissure RN [mm]**	**Commissure LN [mm]**	**Coronary Ostium R [mm]**	**Coronary Ostium L [mm]**
ES w/o sCa	1.62 ± 1.05	1.46 ± 0.72	1.50 ± 0.96	1.96 ± 1.26	1.92 ± 1.47	1.69 ± 1.22	1.72 ± 3.42	1.35 ± 0.91
ED w/o sCa	1.18 ± 0.60	1.38 ± 0.74	1.51 ± 1.00	1.73 ± 1.25	2.16 ± 2.86	1.51 ± 1.09	1.36 ± 2.37	0.99 ± 0.56
ES w/sCa	1.42 ± 0.86	1.36 ± 0.84	1.92 ± 1.42	1.09 ± 0.62	1.71 ± 1.04	1.47 ± 1.35	1.47 ± 1.60	1.06 ± 0.69
ED w/sCa	1.07 ± 0.60	1.91 ± 3.34	1.42 ± 0.91	1.16 ± 0.79	1.49 ± 1.13	1.29 ± 1.12	1.12 ± 1.04	0.99 ± 0.57
Total	1.35 ± 0.84	1.49 ± 1.51	1.56 ± 1.06	1.61 ± 1.16	1.90 ± 1.97	1.53 ± 1.19	1.46 ± 2.54	1.12 ± 0.74

Note: sCa: severe Calcifications, RL mean the commissure point of RCC and LCC, RN mean the commissure point of RCC and NCC LN mean the commissure point of LCC and NCC. Data are mean ± standard deviation for cases.

**Table 2:** Segmentation Results by Dice Coefficient, Mean symmetric Surface Distance and Hausdorff Distance.

	Aortic Root			RCC			LCC			NCC		
	DC	MSD [mm]	HD [mm]	DC	MSD [mm]	HD [mm]	DC	MSD [mm]	HD [mm]	DC	MSD [mm]	HD [mm]
ES w/o sCa	0.96 ± 0.05	0.43 ± 0.93	1.46 ± 3.81	0.70 ± 0.07	0.36 ± 0.13	1.43 ± 1.09	0.67 ± 0.10	0.43 ± 0.17	1.46 ± 1.13	0.69 ± 0.07	0.37 ± 0.12	1.38 ± 1.19
ED w/o sCa	0.95 ± 0.11	0.39 ± 0.49	1.88 ± 4.76	0.71 ± 0.05	0.34 ± 0.12	0.93 ± 0.27	0.70 ± 0.04	0.34 ± 0.07	0.96 ± 0.33	0.69 ± 0.05	0.34 ± 0.08	0.99 ± 0.34
ES w/sCa	0.94 ± 0.09	0.63 ± 1.70	1.80 ± 5.10	0.69 ± 0.06	0.40 ± 0.11	1.00 ± 0.25	0.68 ± 0.08	0.41 ± 0.10	1.01 ± 0.29	0.62 ± 0.08	0.45 ± 0.13	1.25 ± 0.52
ED w/sCa	0.96 ± 0.03	0.52 ± 1.40	1.40 ± 3.88	0.71 ± 0.05	0.36 ± 0.08	0.87 ± 0.16	0.69 ± 0.05	0.37 ± 0.08	0.92 ± 0.19	0.63 ± 0.08	0.43 ± 0.11	1.10 ± 0.27
Total	0.95 ± 0.08	0.46 ± 1.02	1.64 ± 4.36	0.70 ± 0.06	0.36 ± 0.12	1.10 ± 0.70	0.69 ± 0.07	0.38 ± 0.13	1.13 ± 0.85	0.67 ± 0.07	0.39 ± 0.11	1.18 ± 0.77

Note: sCa: severe Calcifications. Data are mean ± standard deviation for cases.

**Table 3:** Segmentation Results by difference of measurements.

Measurement Items		Ground Truth Label Based [mm]	Segmentation Result Based [mm]	Difference [mm]	Absolute Error [mm] (SE)	Error Rate [%] (SE)	CC b/w G and S *r*	Fixed Error*p*-Value	Proportional Error *r_p_*
effective Height	RCC	10.97 ± 2.43	10.54 ± 2.14	−0.43 ± 1.41	1.04 (0.07)	9.47 (0.55)	0.82 *	<0.001 *	−0.22 *
	LCC	11.12 ± 2.44	10.78 ± 2.25	−0.34 ± 1.42	1.05 (0.06)	9.53 (0.63)	0.82 *	<0.001 *	−0.14
	NCC	11.06 ± 2.39	10.56 ± 2.27	−0.50 ± 1.41	1.04 (0.07)	9.17 (0.50)	0.82 *	<0.001 *	−0.09
geometric Height	RCC	14.42 ± 2.40	13.95 ± 2.21	−0.47 ± 1.93	1.40 (0.09)	9.58 (0.55)	0.65 *	<0.001 *	−0.11
	LCC	15.73 ± 2.56	15.24 ± 2.05	−0.49 ± 1.87	1.38 (0.08)	8.99 (0.67)	0.69 *	<0.001 *	−0.30 *
	NCC	16.05 ± 2.47	15.71 ± 2.16	−0.34 ± 1.94	1.39 (0.09)	8.57 (0.48)	0.65 *	0.006	−0.20 *
cusp insertion Length	RCC	42.82 ± 7.13	42.49 ± 6.87	−0.34 ± 4.94	3.80 (0.20)	8.86 (0.42)	0.75 *	0.276	−0.06
	LCC	41.55 ± 6.14	42.05 ± 5.61	0.49 ± 3.92	2.94 (0.16)	7.03 (0.37)	0.78 *	0.044	−0.14
	NCC	42.59 ± 6.53	42.88 ± 6.45	0.29 ± 4.39	3.19 (0.19)	7.41 (0.41)	0.77 *	0.290	−0.02
Free Margin Length	RCC	27.47 ± 5.09	27.62 ± 5.18	0.16 ± 3.19	2.37 (0.13)	8.60 (0.45)	0.81 *	0.431	0.03
	LCC	24.30 ± 4.68	24.46 ± 4.16	0.17 ± 3.19	2.21 (0.14)	9.06 (0.48)	0.75 *	0.394	−0.17
	NCC	25.96 ± 5.20	25.83 ± 4.95	−0.13 ± 3.34	2.42 (0.14)	9.18 (0.50)	0.79 *	0.522	−0.08
commissural Distance	RL-RN	25.62 ± 3.20	25.78 ± 3.23	0.15 ± 1.07	0.84 (0.04)	3.30 (0.16)	0.94 *	0.023	0.02
	RL-LN	23.32 ± 3.12	23.42 ± 3.00	0.10 ± 0.80	0.63 (0.03)	2.76 (0.14)	0.97 *	0.043	−0.16
	LN-RN	23.86 ± 3.26	23.89 ± 3.20	0.04 ± 1.00	0.78 (0.04)	3.33 (0.16)	0.95 *	0.562	−0.05
Nadir Ring Perimeter		89.44 ± 11.59	87.16 ± 11.61	−2.28 ± 8.78	4.39 (0.49)	4.73 (0.59)	0.71 *	<0.001 *	0.002
Commissure Ring Perimeter		114.84 ± 15.56	114.54 ± 17.83	−0.30 ± 8.44	3.31 (0.48)	2.77 (0.37)	0.88 *	0.569	0.28 *
Coronary Height	right	16.62 ± 3.69	16.73 ± 3.58	0.11 ± 1.71	1.22 (0.07)	7.80 (0.53)	0.89 *	0.306	−0.07
	left	14.55 ± 3.05	14.51 ± 2.88	−0.04 ± 1.03	0.77 (0.04)	5.69 (0.35)	0.94 *	0.512	−0.17

Note: RL mean the commissure point of RCC and LCC, RN mean the commissure point of RCC and NCC, LN mean the commissure point of LCC and NCC, CC mean Correlation Coefficient. Data are mean ± standard deviation for cases. * showed *p* < 0.0026.

## Data Availability

The data presented in this study are not publicly available due to restrictions imposed by research ethics.

## Appendix A

Figure A1 shows scatterplots for correlation between the *G*-based values and *S*-based values, for each measurement item. The *G*-based values were plotted on the *x*-axis, and *S*-based values were plotted on the *y*-axis.

Figure A2 shows Bland–Altman plots for each measurement item. Mean of the *G*-based and *S*-based values were plotted on the *x*-axis, and the differences between their values were plotted on the *y*-axis.
